# Trait Specific Expression Profiling of Salt Stress Responsive Genes in Diverse Rice Genotypes as Determined by Modified Significance Analysis of Microarrays

**DOI:** 10.3389/fpls.2016.00567

**Published:** 2016-05-03

**Authors:** Mohammad R. Hossain, George W. Bassel, Jeremy Pritchard, Garima P. Sharma, Brian V. Ford-Lloyd

**Affiliations:** ^1^Department of Genetics and Plant Breeding, Bangladesh Agricultural UniversityMymensingh, Bangladesh; ^2^School of Biosciences, University of BirminghamBirmingham, UK

**Keywords:** transcriptomics, significance analysis of microarrays (SAM), rice, salt tolerance, gene ontology enrichment, regulatory network

## Abstract

Stress responsive gene expression is commonly profiled in a comparative manner involving different stress conditions or genotypes with contrasting reputation of tolerance/resistance. In contrast, this research exploited a wide natural variation in terms of taxonomy, origin and salt sensitivity in eight genotypes of rice to identify the trait specific patterns of gene expression under salt stress. Genome wide transcptomic responses were interrogated by the weighted continuous morpho-physiological trait responses using modified Significance Analysis of Microarrays. More number of genes was found to be differentially expressed under salt stressed compared to that of under unstressed conditions. Higher numbers of genes were observed to be differentially expressed for the traits shoot Na^+^/K^+^, shoot Na^+^, root K^+^, biomass and shoot Cl^−^, respectively. The results identified around 60 genes to be involved in Na^+^, K^+^, and anion homeostasis, transport, and transmembrane activity under stressed conditions. Gene Ontology (GO) enrichment analysis identified 1.36% (578 genes) of the entire transcriptome to be involved in the major molecular functions such as signal transduction (>150 genes), transcription factor (81 genes), and translation factor activity (62 genes) etc., under salt stress. Chromosomal mapping of the genes suggests that majority of the genes are located on chromosomes 1, 2, 3, 6, and 7. The gene network analysis showed that the transcription factors and translation initiation factors formed the major gene networks and are mostly active in nucleus, cytoplasm and mitochondria whereas the membrane and vesicle bound proteins formed a secondary network active in plasma membrane and vacuoles. The novel genes and the genes with unknown functions thus identified provide picture of a synergistic salinity response representing the potentially fundamental mechanisms that are active in the wide natural genetic background of rice and will be of greater use once their roles are functionally verified.

## Introduction

Abiotic stresses greatly reduce agricultural productivity worldwide. The yield of rice, one of the major food crops that feed the world, can be reduced by up to 50% making it highly sensitive to soil salinity. Salinity causes accumulation of excess Na^+^ and Cl^−^ in the shoot which is detrimental for plant nutrition and exerts osmotic stress and ionic toxicity that ultimately restricts plant growth (Munns et al., 2006; Flowers and Colmer, 2008; Munns and Tester, 2008). Salinity tolerance, a complex trait both physiologically and genetically, requires a wide range of physiological and biochemical responses by activating a complex network of genes upon exposure to stress (Cotsaftis et al., 2011; Wu et al., 2013). However, the naturally occurring genetic variation across rice varieties, cultivars, landraces and wild species provides the advantage to identify factors such as genes, proteins and metabolites which can be utilized by conventional breeding and genetic engineering technologies for improvement of crops' tolerance to stresses (Langridge and Fleury, 2011; Horie and Karahara, 2012).

The advances in the high throughput multi-omics techniques along with the progress made in the ever spreading arena of bioinformatics, have given rise to the system biology approaches (Duque et al., 2013). This allows the investigation of the natural genotypic variation holistically to gain deeper biological insight on how the plant functions as a whole by discovering the putative functions of genes, proteins and metabolites in a specific biological context by dissecting the complex regulatory networks of genes, proteins, and metabolites associated with stress adaptation and tolerance (Mochida and Shinozaki, 2011; Shelden and Roessner, 2013).

Recently, there has been a substantial advancements in the technology of whole genome transcriptomic profiling and a number of crop species such as *Arabidopsis* (Kumari et al., 2008), barley (Close and Wanamaker, 2004), maize (Wang et al., 2003), and wheat (Clarke and Rahman, 2005) etc., have been studied extensively. In rice, several microarray platforms were used such as cDNA microarrays (Ueda et al., 2006), NSF 45 K 70-mer oligo microarrays (Senadheera et al., 2009), Affymetrix gene chips (Walia et al., 2005; Walia and Wilson, 2007, 2009; Cotsaftis et al., 2011) and Agilent 44 k microarray (Aya et al., 2011) to study the response of plants toward stresses at transcritpome level.

Stress responsive transcripts, in most of the cases, are usually identified based on comparative and differential transcriptomic expression analysis in two to four genotypes showing extremely contrasting levels of tolerance which results in the findings being genotype specific. This ceases the opportunity to analyze the gradient of transcriptomic responses in diverse genotypes at a time and to correlate those with the changes in morpho-physiological responses to identify the significant transcripts and hence, the molecular functions and biological processes that are operating in species level as adaptive mechanisms (Feder and Walser, 2005; Hossain et al., 2015b). The recent advent of powerful and holistic analytical approaches of system biology such as the Significance Analysis of Microarrays (SAM) offer unique possibility in this regard. And such approaches of correlating the transcriptomic and morpho-physiological responses in a wide genetic background of rice under salt stress are yet to be explored.

SAM was first successfully used to identify the significant changes in transcriptional responses in human lymphoblastoid cells under ionizing radiation with a much lower estimated FDR (12%), compared to the higher FDRs (60–84%) of other conventional methods of analysis (Tusher et al., 2001). This study used a slightly modified version of SAM to suit it with the objective of analyzing the gradient of salinity induced transcriptomic and morpho-physiological responses in diverse genotypes of rice. To achieve this diversity, eight rice genotypes were used that are of diverse geographical origins and belong to different sub-species groups (such as *indica, japonica*, and wild species), different cultivar groups (such as landraces, cultivars, and high yielding varieties) with a reputation of different levels of tolerance to salinity stress (such as susceptible, moderately tolerant, and highly tolerant). Agilent's 44 k oligo-microarrays were used as it provided less expensive yet reasonably sensitive profiling of the genome-wide transcriptional responses of such higher number of samples.

Thus, a modified SAM approach is used to interrogate the patterns of variation in gene expressions by the gradient of responses in morpho-physiological traits in a wide genetic background of rice represented by eight diverse (in terms of taxonomy, origin and salt sensitivity) genotypes in a way to identify the significant transcripts that is relative to the changes in a particular morpho-physiological trait under salt stress. These identified trait specific salinity induced transcripts are discussed along with their complex regulatory networks and the major biological processes and molecular functions that are operating in the wide genetic background of rice as adaptive mechanisms to cope with the stressed environments.

## Materials and methods

### Plant materials

Eight rice genotypes (Table 1) consisting of landraces, cultivars, high yielding varieties (HYVs) and wild species and taxonomically belonging to three different rice sub-species groups namely, *Indica, Japonica*, and wild species, having diverse geographical origin and showing different levels of tolerance to salt stress were obtained from the International Rice Gene Bank Centre (IRGC) of the International Rice Research Institute (IRRI).

**Table 1:** **List of diverse rice genotypes along with their sup-species levels, origins, and reputation of salt stress used for trait specific expression profiling of salinity induced transcripts by modified Significance Analysis of Microarrays (SAM)**.

**Genotype**	**Germplasm Group**	**Accession Number**	**Origin**	**Salt Tolerance status (Hossain et al., 2015a)**
Pokkali	*Indica* (landrace)	IRGC 108921	India	Tolerant
PSBRc50	*Indica* (variety)	IRGC 99706	Philippines	Moderately tolerant
IR 58	*Indica* (variety)	IRGC 63492	Philippines	Moderately tolerant
BRRI dhan 29	*Indica* (HYV)	IRTP 15241	Bangladesh	Susceptible
Banikat	*Japonica* (cultivar)	IRGC 67720	India	Moderately tolerant
Nipponbare	*Japonica* (cultivar)	IRGC 117274	Japan	Susceptible
*O. latifolia*	Wild species	IRGC 100965	Costa Rica	Susceptible
*O. rufipogon*	Wild species	IRGC 105390	Thailand	Susceptible

*HYV, High yielding variety, IRGC, International Rice Germplasm Center, IRTP, International Rice Testing Program*.

### Hydroponic culture conditions and salinity treatments

**Figure 1:**
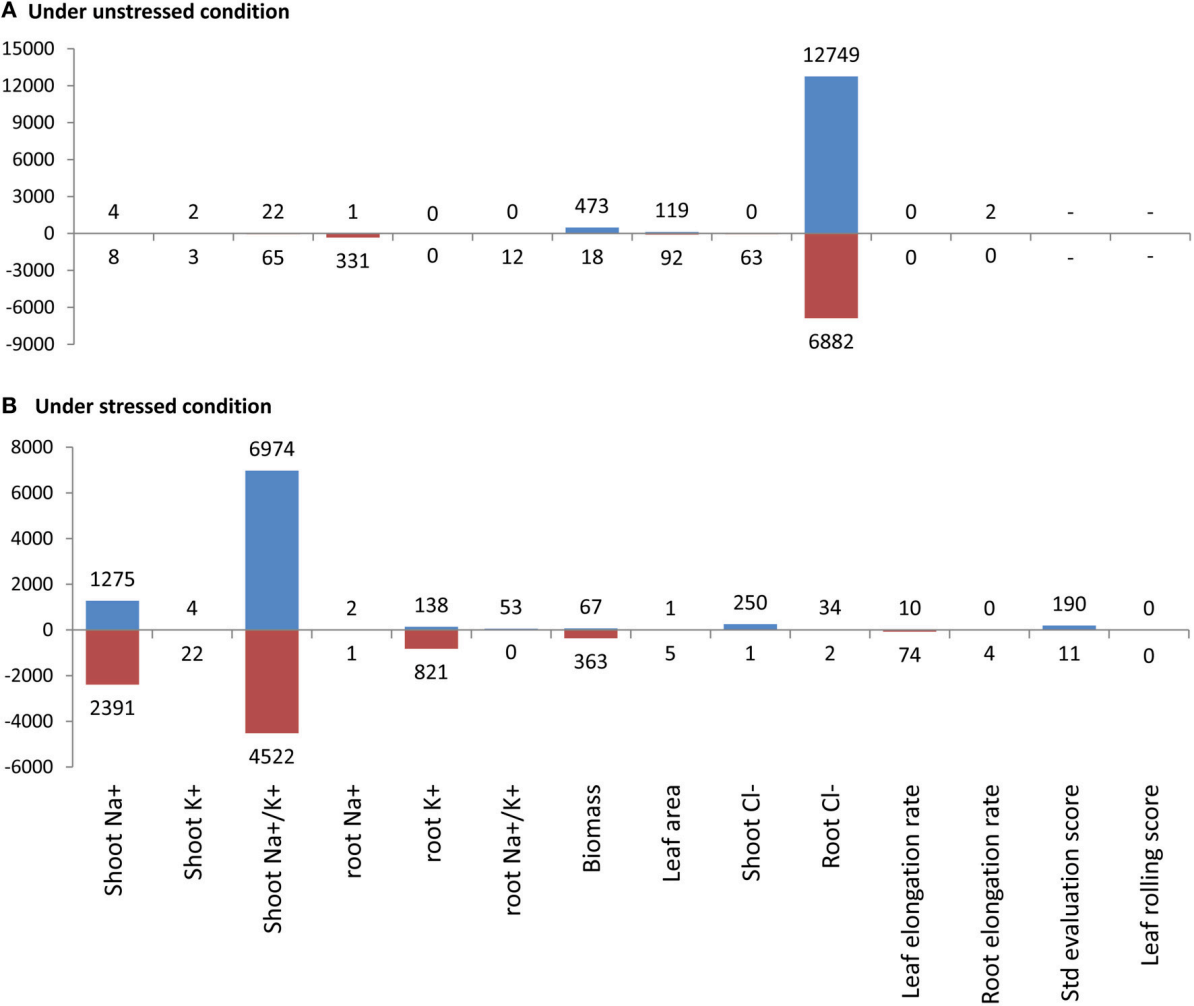
**Number of significant positively (blue bars) and negatively (red bars) expressed probes for each of the 14 morpho-physiological traits in the eight rice genotypes representing wide natural variation under unstressed (A) and stressed (B) conditions as determined by the modified SAM approach**. The complete lists of the significant probes along with the corresponding fold score (*d*), *q*-value and functional annotation can be found in Tables S1a–n, S2a–n.

Plants were grown twice maintaining the same environmental conditions in growth room. In the first growing, the seedlings in the flasks were challenged with 80 mM NaCl at 14 days after seedling emergence (DAE) and the data for the 14 morpho-physiological traits were collected within the next 7 days. The details of the traits (names of the traits are shown in Figure 1) can be found in Hossain et al. (2015a). In the second growing, 120 mM NaCl stress was used and total RNA from the whole seedlings was extracted after 48 h of stressed period (i.e., at 16 DAE) for whole genome transcriptome profiling. The details of the plant growth conditions, salinity treatments, and the collection of morpho-physiological data can be found in Hossain et al. (2015a).

### Extraction and quality assessment of total RNA

The total RNA was extracted from the 16 days old seedlings using the QIAGEN RNeasy Plant Mini Kit. For seedlings weighing more than the recommended amount of starting material (100 mg), the sections of root, stem, and leaves were used to represent the whole seedlings. Each sample from each individual seedling represented a replicate. The freshly harvested samples were snap frozen in liquid nitrogen and were immediately ground to a fine powder with pre-chilled mortar and pestle. The RNA was purified using an RNeasy spin column and an on-column DNase treatment. The total RNA was eluted in 30 μl of RNase free water and was immediately aliquoted and stored in a −70°C freezer. Only the eluted total RNA having the 260/280 and 260/230 ratios around 2.0 (as quantified by Nanodrop ND-1000 VIS spectrophotometer v. 3.2.1) and having the RNA integrity (RIN) value >7.0 (as detected by the Agilent 2100 bioanalyser using the RNA 6000 Nano Labchip kit) were used hybridization.

### Microarray hybridization, feature extraction, and rice genome array

The 48 extracted RNA samples were then prepared for microarray hybridization in the Genomics Services Facility of the University of Birmingham where cDNA synthesis, preparation of One-Color Spike-Mix, Cy3 labeling, microarray hybridization on Agilent 4 × 44 K rice oligo microarrays, scanning and feature extraction was carried out using Agilent's one color Quick Amp labeling kit (v.6.5, May 2010). The array represented about 43,803 rice genes with one 60-mer oligonucleotide probe representing each and consists of 32,325 probes corresponding to RAP loci with full-length representative cDNA, 6943 probes to RAP loci with EST support, 2612 probes to gene predicted loci and control probes including false positives and non-rice sequences etc. All microarray data of this work is available from the NCBI-GEO website (http://www.ncbi.nlm.nih.gov/geo/) under GEO Series entry GSE79043.

### Modified significance analysis of microarrays (SAM)

The significance analysis of microarrays (http://statweb.stanford.edu/~tibs/SAM/) was used to identify the salinity induced, trait specific significant transcripts in diverse rice genotypes. The basic principle of SAM includes scoring each of the transcripts based on the changes in both gene expression and response variable. And it uses repeated permutations of the data to determine if the change in the expression of any gene is significantly related to the changes in response variable.

This study adopted a little modification as it analyzed the control and treated data separately. Unlike the unstressed condition, the stressed condition rendered substantial amount of variation in both gene expression and morpho-physiological trait responses in the eight diverse rice genotypes. So, it was only sensible to analyze the treated data separately (which otherwise is analyzed together in a mono-genotypic study) allowing SAM to identify the stress inducible significant transcripts.

For a particular trait (e.g., for shoot Na) under stress condition, firstly the pre-processed (quantile normalized and log_2_ transformed) gene expression data of all the treated samples is arranged in a single spreadsheet (transcripts in rows; genotypes in column). The names of the samples (such as genotype-1, replication-1) in column heads are replaced by their corresponding trait responses which is weighted to a scale of 0 to 1. The SAM program is then ran using the response type as “quantitative” based on 100 permutations using SAM version 4.0 software. The significant genes (relative to shoot Na, in this instance) are selected based on the combined criteria of ≤ 5% FDR and ≥2-fold score. To analyze the same for the rest of the traits, the sample names in the original spreadsheet is replaced by the weighted ranks (0–1) of the trait in question. Further details of the SAM procedure used in this study can be found in Hossain (2014) and in SAM manual.

### Gene ontology (GO) enrichment analysis

The list of significant positively and negatively expressed genes were subjected to GO analysis using the AgriGO web-based tool (Du et al., 2010), available from http://bioinfo.cau.edu.cn/agriGO/index.php. Singular Enrichment Analysis (SEA) (Huang et al., 2009) was used to identify the biological processes or the molecular functions that are significantly enriched by the identified positively or negatively expressed genes for each of the traits. “Rice TIGR gene model” were used as reference during SEA.

### Mapping of the genes on chromosomes and the regulatory network of genes

The list of probes was mapped in the 12 chromosomes of rice using the Chromosome Map Tool (http://viewer.shigen.info/oryzavw/maptool/MapTool.do) of GRAMENE genome browser database. The interactions between all the significant probes, as determined by SEA, were determined using the “The Rice Interactions Viewer” web based tool version Interactome 2.0 developed by the Bio-Analytic Resource- the BAR that queries a database of 37472 predicted and 430 confirmed Rice interacting proteins (http://bar.utoronto.ca/welcome.htm). Prior to using the “Rice Interactions Viewer,” the RAP-DB gene Ids (e.g., Os06g0699400) were converted into MSU (TIGR) ID (e.g., LOC_Os06g48590) using the RiceXPro: Global gene expression profile web based tool (as shown in Table S4d).

## Results and discussion

The gene expression data of eight rice genotypes were interrogated with their morpho-physiological data using modified SAM approach to identify the trait specific gene expression pattern in the wide genotypic background of rice under both stressed and unstressed conditions for each of the 14 morpho-physiological traits. The detailed morpho-physiological data (not shown here) are published as a separate article (Hossain et al., 2015a).

### Globally, more genes are expressed under salt stress

In general, higher numbers of probes were found to be significantly expressed, both positively and negatively for most of the important salinity tolerance related traits *viz*., shoot Na^+^, shoot Na^+^/K^+^, Biomass, root K^+^, and shoot Cl^−^ under salt stress compared to that of unstressed conditions (Figure 1; Tables S1, S2). For example, 1275 and 2391 probes were positively and negatively expressed, respectively for shoot Na^+^, the single most important parameter to study salinity tolerance, under stressed condition compared to only 4 and 8 probes being positively and negatively expressed, respectively under unstressed conditions. Interestingly a very high number of genes were negatively expressed under stressed conditions, compared to that of under unstressed condition, for the trait “biomass” (Figure 1; Tables S1g, S2g, S3). All these probably indicate that plants deploy their adaptive mechanism by differentially activating a large number of genes under stressed conditions which otherwise is not activated constitutively.

Nonetheless, it is to keep in mind that these results are obtained by studying the RNA extracted from the whole seedling. And as gene expression, recently, is known to vary depending on cell or tissue types which can even be in opposite directions in some cases (Taylor-Teeples et al., 2011; Otsuki et al., 2014), verification of expression of all the identified genes in each cell or tissue types via *in situ* analysis will be helpful in revealing their exact roles in salinity tolerance mechanism.

### Genes involved in ion homeostasis and transport

Lists of significant genes were mined manually to identify the putative genes that might be involved in ion transport with particular attention being given to the genes that were found to be significant for shoot Na and shoot Na/K. In total, 60 genes were found to be involved in ion homeostasis and transport processes (Table 2). Among the genes involved in sodium homeostasis, the notables are Na^+^/H^+^ exchangers such as Os09g0286400, Os05g0382200, Os11g0648000, and Os12g0641100 and potassium transport related genes such as Os03g0656500, Os07g0102100, Os03g0337500, Os01g0932500, Os02g0519100, Os03g0575200, and Os04g0682800 etc. Genes for other cations such as Ca^2+^, Mg^2+^ and anions such as ammonium, nitrate, sulfate, and phosphate were also significantly expressed under salt stress.

**Table 2:** **Lists of genes (among the significant genes for (a) shoot Na^+^ and (b) shoot Na^+^/K^+^) involved in ion homeostasis and transports under salinity stress in wide natural variation of rice genotypes**.

**Probe Name**	**Annotation**	**(a) among the significant genes for shoot Na^+^**		**(b) among the significant genes for shoot Na^+^/K^+^**	
		**Fold score**	***q*-value (%)**	**Fold score**	***q*-value (%)**
Os01g0557500	Cation/proton exchanger 1a.	−2.48	3.03		
Os01g0645200	Bile acid:sodium symporter family protein.	−2.35	3.35		
Os05g0382200	Na+/H+ exchangeing protein-like.			2.02	3.29
Os06g0152200	Salt-tolerance protein.	−2.11	4.88		
Os06g0701600	Cation transporter family protein.	−2.96	1.30		
Os08g0503700	Sodium/sulfate symporter family protein.			−2.26	2.81
Os09g0286400	Sodium/hydrogen exchanger family protein.			2.25	2.34
Os09g0299400	Sodium-and chloride-activated ATP-sensitive potassium channel.			−2.72	1.79
Os09g0484900	Sodium-dicarboxylate cotransporter-like.			−2.01	3.89
Os10g0436900	Sodium/calcium exchanger membrane region domain cont. protein.	−2.13	4.88		
Os11g0648000	Sodium/hydrogen exchanger subfamily protein.			2.13	2.81
Os12g0170300	Bile acid:sodium symporter family protein.			2.20	2.34
Os12g0641100	Sodium/hydrogen exchanger family protein.			−2.64	1.79
Os01g0210700	Potassium channel (Fragment).	−2.08	4.88		
Os01g0369300	Potassium transporter 1 (AtPOT1) (AtKUP1) (AtKT1).	−2.11	4.88		
Os01g0648000	Potassium channel.			2.64	1.36
Os01g0696100	K+ channel, two pore family protein.			2.33	1.79
Os01g0932500	K+ potassium transporter family protein.			2.65	1.36
Os02g0519100	K+ potassium transporter family protein.			2.40	1.79
Os02g0612700	K+ channel tetramerisation domain containing protein.	−2.52	2.51		
Os03g0337500	K+ potassium transporter family protein.	−3.57	0.71		
Os03g0575200	K+ potassium transporter family protein.			1.88	3.89
Os03g0656500	K-exchanger-like protein.	−2.24	3.62		
Os04g0401700	Potassium transporter 5 (AtPOT5) (AtHAK1) (AtHAK5).			−2.15	3.29
Os04g0682800	Potassium efflux system protein family protein.			−2.28	2.81
Os06g0625900	Potassium transporter 8 (AtPOT8) (AtHAK8).	−2.94	1.44		
Os06g0671000	Potassium transporter 1 (AtPOT1) (AtKUP1) (AtKT1).			−2.22	2.81
Os07g0102100	K+ potassium transporter family protein.	2.85	1.92		
Os07g0669700	Potassium transporter 4 (AtPOT4) (AtKUP3) (AtKT4).	−3.17	0.93		
Os01g0678500	Two-pore calcium channel.	−2.15	4.88		
Os01g0908500	Mg2+ transporter protein, CorA-like family protein.	−2.13	4.88		
Os02g0138900	Low affinity calcium antiporter CAX2.	−2.54	2.51		
Os02g0720700	Cl− channel, voltage gated family protein.			1.99	3.29
Os04g0605500	Calcium-transporting ATPase 8, plasma membrane-type	−2.93	1.44		
Os04g0653200	Low affinity calcium transporter CAX2 (Fragment).			1.88	3.89
Os05g0594200	Calcium/proton exchanger superfamily protein.	2.65	3.03		
Os03g0150800	High affinity phosphate transporter 2 (Phosphate transporter).			2.91	0.88
Os03g0161200	Sulfate transporter 3.1 (AST12) (AtST1).	−2.95	1.30		
Os03g0195800	High affinity sulfate transporter.			3.62	0.38
Os03g0838400	Ammonium transporter.	−2.99	1.30		
Os04g0185600	Phosphate transporter 6.	−3.65	0.71		
Os05g0477800	High-affinity sulfate transporter HvST1.			2.69	1.09
Os08g0155400	Nitrate transporter (Fragment).	−2.67	2.15		
Os08g0406400	Sulfate transporter (Fragment).	2.67	2.51		
Os09g0240500	Sulfate transporter 4.1, chloroplast precursor (AST82).	−2.26	3.62		
Os10g0444600	Phosphate transporter (Fragment).	−2.50	2.51		
Os01g0588200	Voltage-dependent anion channel.			2.78	1.09
Os01g0704100	Membrane transporter.	−2.52	2.51		
Os01g0975900	Tonoplast membrane integral protein ZmTIP1-2.	3.31	0.80		
Os02g0117500	Glutamate receptor 3.2 precursor (AtGluR2).			3.38	0.39
Os02g0255000	Cyclic nucleotide-gated ion channel 1 (AtCNGC1)			2.09	2.81
Os02g0823100	Plasma membrane intrinsic protein (ZmPIP1-5)	−2.61	2.15		
Os03g0129100	Seven transmembrane protein MLO2.	−2.78	1.92		
Os03g0758300	Cyclic nucleotide-gated ion channel 2 (AtCNGC2)	−2.35	3.35		
Os04g0643600	Cyclic nucleotide-gated channel C (Fragment).	−2.13	4.88		
Os05g0231700	Tonoplast membrane integral protein ZmTIP4-2.	3.35	0.67		
Os06g0527400	Cyclic nucleotide-gated calmodulin-binding ion channel.			1.84	3.89
Os08g0555000	Transmembrane 9 superfamily protein member 2 precursor (p76).	2.83	1.92		
Os09g0541000	Plasma membrane intrinsic protein 2c, (PIP2c, TMP2C)	2.56	3.35		
Os12g0639800	Vesicle-associated membrane protein 722 (AtVAMP722)	−2.70	1.92		

Several membrane intrinsic and ion channel related genes having putative roles in ion homeostasis were also found to be significantly expressed e.g., aquaporin (Os09g0541000); membrane transporter (Os01g0704100), tonoplast integral protein (Os01g0975900, Os05g0231700); vesicle associated membrane protein (Os12g0639800), and ion channels (Os04g0643600, Os06g0527400, Os02g0255000, Os03g0758300, Os02g0117500, and Os01g0588200) etc. The cation transporter family protein (Os06g0701600) and cation/proton exchanger (Os01g0557500) are found to be negatively expressed for shoot Na.

Around 15 genes such as Os01g0290800, Os01g0356000, Os01g0609200, Os01g0609300, Os01g0966100 etc., were found to be involved in ABC transport (not shown in table). The ABC-transporter proteins are believed to transport various substrates such as ions, amino acids, sugars and peptides across cellular membranes besides their role in detoxification, plant growth and developmental processes (Martinoia et al., 2002; Davidson et al., 2008). In yeast, ABC transporters are found to be involved in cation homeostasis but their role in plants is yet to be identified (Rea, 1999; Kang et al., 2010).

### Global regulation of biological processes (BP) under salt stress

More biological processes (BP) are significantly enriched by the induced genes (Table 3; Tables S4b,c) compared to that of by the constitutive genes (Tables S5b,c) as determined by the Singular Enrichment Analysis (SEA). Under unstressed conditions, no BPs were significantly enriched by the genes that are expressed for shoot Na^+^ and shoot Na^+^/K^+^ (Table S5b1), whereas most of the BPs were enriched by the genes that are expressed for these tissue ion traits under stressed conditions (Table 3; Table S4b1). This clearly shows that salt stress activates a series of genes which enrich different BPs in response to the stress across the range of genotypes. The major BPs that are activated under stressed conditions are Apoptosis, Stress Response, Signaling process, Transport, Metabolic and Catabolic process, Cellular, and Developmental processes etc (Table 3). The role of individual genes is not described in detail in this section, instead is discussed according to the molecular functions enriched by these genes in the next section.

**Table 3:** **List of significant GO categories of biological process under stressed condition for significant positively and negatively expressed genes for different morpho-physiological traits as determined by Singular Enrichment Analysis**.

**GO Category: Biological Processes**		**Positively Expressed Genes**			**Negatively Expressed Genes**		
		**Shoot Na^+^**	**Shoot Na^+^/K^+^**	**Root K^+^**	**Shoot Na^+^**	**Shoot Na^+^/K^+^**	**Root K^+^**
Apoptosis	Programmed cell death			9			
Stress Response	Response to abiotic stimulus		29		12	29	
	Response to chemical stimulus (response to endogenous, organic substance, and hormone)		110		19	77	6
	Response to biotic stimulus	8	14			11	
	Cellular response to stimulus		77			46	
Signaling process	Signal transduction, intracellular signaling process; signaling pathway		125				
Transport	Transmembrane transport					35	
	Di-, tri-valent inorganic cation transport; and transition metal ion transport				9	13	
Metabolic processes	Regulation of transcription, gene expression				177		
	Negative regulation of gene expression (silencing)					9	
	Translation	45					
	Cellular nitrogen compound biosynthetic process (amine, amino acid biosynthetic process)					61	
	Protein modification by small protein conjugation or removal		31		19	22	
	Protein amino acid dephosphorylation				16		
	Generation of precursor metabolites and energy (including photosynthesis, light harvesting)		26		43	67	
	Small molecule metabolic process		15			208	
	Cellular lipid metabolic process					51	
	Cellulose metabolic process		17				
	Secondary metabolic process		31		11	34	
Catabolic process	Including protein, polysaccharadie catabolic process		126				
Cellular process	Cell cycle		17		7	23	
	DNA conformation change (DNA packaging)	16					
	DNA recombination		32				
	Microtubule cytoskeleton organization		6				
	Cellular macromolecular complex subunit organization	19					
Developmental process	Multicellular organismal process	8	88		27	31	10
	Cellular cell wall organization or biogenesis		70		9	13	
	Reproduction		66		18		
	Regulation of anatomical structure size		9				
	Oxidation reduction	29		6	54		12

*The details (GO terms, p-values, FDR values, GO flash charts and schematic diagrams) can be found in Tables S4b1,2*.

### Global regulation of molecular functions (MF) under salt stress

Salt stress significantly enriches more molecular functions (MF) in the wide genetic background of rice compared to unstressed conditions as determined by the SEA (Table 4; Tables S4a,c, S5a,c). Significant positively and negatively expressed transcripts under salt stress enriched a number of MFs that includes signal transducer activity, transcription, and translation factory activity, serine hydrolase and metalloexopeptidase activity etc., (Table 4). The individual genes of important molecular functions are discussed below and the detailed discussion of all MFs can be found in Hossain (2014).

**Table 4:** **List of significant GO categories of molecular function under stressed condition for significant positively and negatively expressed genes for different morpho-physiological traits as determined by Singular Enrichment Analysis**.

**GO category: Molecular Functions**		**Positively expressed genes**			**Negatively expressed genes**			
		**Shoot Na^+^**	**Shoot Na^+^/K^+^**	**Root K^+^**	**Shoot Na^−^**	**Shoot Na^+^/K^+^**	**Root K^+^**	**Biomass**
Signal transducer activity			107			56		
Binding	Transcription factor activity				81			
	Translation factor activity (nucleic acid binding)		36			26		
	SNAP receptor activity					6		
	Chaperone binding					6		
	Manganese ion binding				18	18		
	Alkali metal ion binding (including potassium ion binding)		8					
	2 iron, 2 sulfur cluster binding					5		
Catalytic activity	Phosphoprotein phosphatase activity (including protein serine/threonine phosphatase activity)				21			
	Protein methyltransferase activity		13					
	Serine O-acyltransferase activity (including serine O-acetyltransferase activity)				5			
	Serine hydrolase activity (including endopeptidase activity)	10	55			25		5
	Metalloexopeptidase activity		16			9		
	Oxidoreductase activity, acting on CH-OH group of donors					39		
	Oxidoreductase activity, acting on single donors with incorporation of molecular oxygen					12		
Electron carrier activity		27	125	6	48	109	17	

*The details of the GO terms, p-values, FDR values, GO flash charts and schematic diagrams can be found in Tables S4a1,a2,c*.

### Signal transducer activity

Stress is first sensed by the receptors in membranes, which then generates secondary signal messengers like calcium, reactive oxygen species, kinases, and phosphates followed by the activation of transcription factor genes that eventually coordinates the plant's adaptive biochemical and physiological responses (Huang et al., 2012; Proietti et al., 2013). This study indentified 107 up-regulated and 54 down-regulated transcripts that are involved in signaling (Table 5).

**Table 5:** **Lists of positively (a) and negatively (b) expressed significant transcripts (for shoot Na^+^/K^+^) that are involved in the Molecular Function “Signal transducer activity” as determined by the Singular Enrichment Analysis (SEA)**.

**Name**	**Annotation**
**(a) Positively expressed transcripts (107)**	
Os08g0442700, Os07g0134200, Os06g0334300	Receptor-like kinase, Receptor-like protein kinase 3
Os07g0107800	Phytosulfokine receptor precursor (EC 2.7.1.37)
Os05g0155200, Os07g0259100, Os03g0701700, Os04g0169100, Os02g0820900	Ethylene receptor, Ethylene receptor homolog, Ethylene receptor-like protein 2
Os07g0132500	Lectin-like receptor kinase 7;2
Os01g0239700, Os08g0446400, Os01g0140400	Leucine-rich receptor-like protein kinase
Os01g0836800	Lung seven transmembrane receptor family protein
Os07g0522600	Metabotropic γ-aminobutyric acid receptor, type B family protein
Os02g0131600	Mitochondrial import receptor subunit TOM22, TOM9 homolog
Os02g0117500	Glutamate receptor 3.2 precursor (AtGluR2)
Os02g0245100	Peroxisomal targeting signal type 2 receptor
Os06g0225300	Brassinosteroid insensitive 1-associated receptor kinase 1 precursor
Os01g0665200	Mitogen-activated protein kinase, Blast and wounding induced
Os06g0699400	MAP kinase 2
Os05g0576800	MAP kinase homolog
Os06g0154500	Mitogen-activated protein kinase (MAP kinase 6)
Os10g0533600	Mitogen-activated protein kinase homolog MMK2 (EC 2.7.1.37)
Os09g0349800, Os09g0349600, Os08g0493800, Os04g0540900, Os02g0111800, Os06g0693200, Os02g0153200, Os04g0658700, Os03g0791700, Os05g0525600, Os08g0203700, Os02g0151100, Os01g0976900, Os10g0155800, Os01g0960400, Os10g0497600, Os01g0664200, Os01g0110500, Os01g0741200, Os02g0218400, Os02g0227700, Os02g0153100, Os03g0148700, Os06g0693000, Os01g0514700, Os01g0114900, Os01g0738300 Os05g0414700, Os06g0654600	Protein kinase domain containing protein
Os10g0533800, Os07g0131100, Os03g0772600, Os12g0562500	Protein kinase family protein
Os01g0323000, Os01g0631700, Os10g0136400 Os07g0537200	Serine/threonine kinase, Ser Thr specific protein kinase-like protein
Os01g0223900, Os02g0527900	Curculin-like (mannose-binding) lectin domain containing protein
Os02g0150800	Cyclin-like F-box domain containing protein
Os12g0256000, Os05g0407500	Esterase/lipase/thioesterase domain containing protein
Os07g0613300	Exportin-t
Os03g0284100	Expressed protein (Pseudo-response regulator 9) (Timing of CAB expression 1-like protein)
Os03g0637600	Extensin protein-like
Os08g0332800	F7O18.23 protein (SWP1) (Struwwelpeter 1 protein)
Os08g0230300	Galactose oxidase, central domain containing protein
Os06g0199800	GPCR, family 2, secretin-like protein
Os06g0111400	Guanine nucleotide binding protein, alpha subunit family protein
Os06g0163000	Heat shock protein STI (Stress inducible protein) (GmSTI)
Os01g0923700	Histidine kinase-like protein
Os01g0114700	LRK33
Os07g0584200, Os04g0477000	NPH3 domain containing protein
Os06g0625300	Peptidoglycan-binding LysM domain containing protein
Os06g0687800	Pincher
Os07g0130700	Resistance protein candidate (Fragment)
Os08g0376700, Os02g0618200, Os06g0654300, Os09g0532400, Os03g0224200	Response regulator 1, Response regulator receiver domain containing protein
Os07g0537900	SRK3 gene
Os05g0525000	TMK protein precursor
Os01g0904700, Os06g0183100	Two-component response regulator ARR1. Splice isoform 2
Os06g0574200, Os02g0218600	UspA domain containing protein
Os09g0416700	Vesicle transport v-SNARE family protein
Os02g0205400	WD40-like domain containing protein
Os02g0830200, Os04g0524300	ZmRR2 protein (Response regulator 2)
Os05g0112000, Os01g0974400, Os03g0275300	Zn-finger, RING domain containing protein
Os06g0716000, Os04g0433600	Protein of unknown function DUF668 family protein
Os09g0573200, Os09g0470900	Conserved hypothetical protein
Os03g0738800, Os07g0501800, Os04g0631900, Os01g0690900	Hypothetical protein
**(b) Negatively expressed transcripts (54)**	
Os10g0346600	BP-80 vacuolar sorting receptor
Os11g0473000	ER lumen protein retaining receptor (HDEL receptor)
Os05g0529300	ER lumen protein retaining receptor (HDEL receptor)
Os06g0680500	Glutamate receptor 3.1 precursor (Ligand-gated ion channel) (*AtGLR2*)
Os06g0717200	Leucine-rich repeat/receptor protein kinase precursor
Os11g0514500	Leucine-rich repeat-containing extracellular glycoprotein precursor
Os03g0343400	Photolyase/blue-light pthotoreceptor (PHR2)
Os01g0176400	Photoreceptor-interacting protein-like
Os01g0114600	Receptor-like kinase ARK1AS (Fragment)
Os06g0496800	Serine/threonine kinase receptor precursor
Os08g0480100	Signal recognition particle receptor protein (Fragment)
Os05g0100700, Os08g0174700	Somatic embryogenesis receptor kinase-like protein
Os06g0708000	MAP kinase homolog
Os06g0367900	Mitogen-activated protein kinase homolog
Os05g0566400	Mitogen-activated protein kinase. Blast and wounding induced
Os01g0206800, Os08g0203400, Os05g0588300, Os05g0258400, Os05g0480400, Os02g0228300, Os01g0116400, Os06g0676600, Os02g0821400, Os01g0779300, Os02g0106900	Protein kinase domain containing protein
Os11g0678000, Os01g0114100	Protein kinase family protein
Os01g0121100	AR401
Os01g0958100	Cell division transporter substrate-binding protein, FtsY family protein
Os03g0284100	Expressed protein (Pseudo-response regulator 9) (Timing of CAB expression 1-like protein)
Os03g0637600	Extensin protein-like
Os07g0535700	F-box protein interaction domain containing protein
Os06g0111400	Guanine nucleotide binding protein (G-protein), alpha subunit family protein
Os11g0206700	Guanine nucleotide binding protein (G-protein)
Os05g0186100	Histidine-containing phosphotransfer protein 4
Os01g0855600	Hs1pro-1 protein
Os02g0259100	Hypothetical protein
Os02g0459600	Legume lectin, beta domain containing protein
Os11g0102200	NPH1-1
Os03g0206700, Os12g0117600, Os11g0118300, Os03g0347700, Os11g0118500	NPH3 domain containing protein
Os05g0127200	Phosphoinositide-specific phospholipase C (PLC) family protein
Os07g0694000	Phosphoinositide-specific phospholipase C
Os07g0695100	Response regulator receiver domain containing protein
Os02g0729400	Rhodanese-like domain containing protein
Os12g0117400	RPT2-like protein
Os11g0143300	Type-A response regulator
Os01g0560200, Os01g0707300	Vesicle transport v-SNARE family protein

*The chromosomal distribution of the positively expressed genes is shown in Figure S1*.

Transcripts of three receptor like kinases (RLK) such as Os08g0442700, Os07g0134200, and Os06g0334300 are up-regulated. RLKs are known to play roles in stress defense by sensing the extracellular signals and activating downstream pathways by phosphorylating target proteins (Marshall et al., 2012; Tanaka et al., 2012). Very recently, in rice, a putative RLK gene, *OsSIK1*, with extracellular leucine-rich repeats (Ouyang et al., 2010) and a cysteine-rich repeat (CRR) RLK sub-family gene, *ARCK1* (Tanaka et al., 2012) was reported to be induced by salt and drought stresses.

Transcripts of 5 Ethylene receptor genes such as Os05g0155200, Os07g0259100, Os03g0701700, Os04g0169100, and Os02g0820900 were found to be positively expressed under salt stress. The role of ethylene in salt stress response is reported to be equivocal (Achard et al., 2006) and In tobacco, an ethylene receptor gene, *NTHK1* was reported to promote leaf growth (Cao et al., 2006), which demonstrated the significance of these ethylene receptor genes to be studied further in determining their role in salt tolerance.

Among the leucine-rich repeat containing transcripts, three (Os01g0239700, Os08g0446400, and Os01g0140400) and two (Os06g0717200 and Os11g0514500) were found to be negatively regulated in this study, respectively. Cheng et al. (2009) reported a leucine-rich-repeat type receptor-like protein kinase, *OsRPK1* to be induced by multiple stresses in plasma membrane of cortex cells in rice roots and Lorenzo et al. (2009) reported an increase in expression of leucine-rich gene, *Srlk* in *Medicago truncatula* roots upon exposure to salt stress. The *Srlk* gene also had a homolog, Os05g0414700, which was also found to be positively expressed in this study.

Protein kinases regulate the phosphorylation and dephosphorylation of other proteins and play a crucial role in stress signal transduction. To date, several plant protein kinases, particularly the mitogen-activated protein (MAPK/MPK) kinase are reported to be activated by osmotic stress (Fujita et al., 2006; Sinha et al., 2011). Five MAP kinases including MAP kinase 2 (Os06g0699400), 6 (Os06g0154500) and MAPK homolog MMK2 (Os10g0533600) were positively regulated and three MAP kinases (Os06g0708000, Os06g0367900, and Os05g0566400) are negatively expressed in wide natural rice variation (Table 5). In addition, Serine/threonine protein kinases have also been known to be involved in multi-stress tolerance in plants (Zhao et al., 2009). Among the serine/threonine kinases, four (Os01g0323000, Os01g0631700, Os10g0136400, and Os07g0537200) was positively expressed and only one (Os06g0496800) was found to be negatively regulated. At least, 33 and 13 protein kinase domain containing proteins were found to be positively and negatively expressed in the wide natural variation of rice genotypes.

Among the many other positively expressed transcripts, notable are heat shock protein, mannose binding, extensins, histidin kinases, NPH3 domain containing protein, and Zn-finger domain containing proteins. These transcripts were found to be located in all the chromosomes except in chromosome 11 and chromosomes 1, 2, 6, and 7 contained most of the genes (Figure S1).

### Transcription factor (TF) activity

The rice genome is known to have 1772 TFs that generally falls under the family of WRKY, DREB, CBF, MYB, bZIP, ERF, zinc-finger, helix-loop-helix, and NAC (Sreenivasulu and Miranda, 2004; Duque et al., 2013). Of the negatively expressed transcripts for shoot Na^+^/K^+^, 81 transcripts were found to significantly enrich the “Transcription factor activity” (Table 6 and Table S4a2). The four bZip family TFs (Os01g0542700 encoding *OsbZIP4*, Os03g0770000 encoding *OsbZIP32*, Os08g0543900 encoding *OsbZIP68*, and Os11g0154800) that were found to be salt responsive in this study have not been reported before. Some of the identified salt and drought responsive bZIP proteins are *OzBZ8* (Mukherjee et al., 2006), *OsbZIP15* (Zou et al., 2008), *OsbZIP23* (Xiang et al., 2008), *OsbZIP46* (Tang et al., 2012), *OzAREB1* (Jin et al., 2010), and *OsbZIP16* (Chen et al., 2012).

**Table 6:** **Lists of significant transcripts (for shoot Na^+^/K^+^) that are involved in transcription factor activity as determined by the Singular Enrichment Analysis (SEA)**.

**Name**	**Annotation**
Os01g0952800	Basic helix-loop-helix dimerisation region bHLH domain containing protein
Os01g0542700, Os03g0770000	Basic-leucine zipper (bZIP) transcription factor domain containing protein
Os08g0543900	BZIP transcription factor RF2b
Os11g0154800	DNA-binding factor of bZIP class (Fragment)
Os01g0826400	WRKY transcription factor 24
Os01g0750100	WRKY transcription factor 31
Os02g0770500	WRKY transcription factor 32
Os01g0665500, Os07g0583700	WRKY transcription factor 34
Os02g0265200	WRKY transcription factor 39
Os02g0462800	WRKY transcription factor 42 (Transcription factor WRKY02)
Os08g0276200	WRKY transcription factor 44 (WRKY DNA-binding protein 44)
Os01g0730700	WRKY transcription factor 49
Os03g0798500	WRKY transcription factor 6
Os06g0158100	WRKY transcription factor 63
Os02g0181300	WRKY transcription factor 71 (Transcription factor WRKY09)
Os03g0680800	BEL1-related homeotic protein 14 (Fragment)
Os03g0762000	Casein kinase II alpha subunit
Os06g0127100	CBF-like protein
Os02g0203000	Concanavalin A-like lectin/glucanase domain containing protein
Os04g0597300	DNA-binding WRKY domain containing protein
Os01g0678700	DP protein
Os01g0165000	DRE binding protein 2
Os03g0152100	E2F dimerization factor
Os02g0537500	E2F homolog
Os04g0669200	Ethylene response factor 3
Os05g0497200	Ethylene responsive element binding factor 4 (AtERF4)
Os05g0497300, Os06g0194000	Ethylene responsive element binding factor 5 (AtERF5)
Os02g0655200	Ethylene responsive element binding factor3 (OsERF3)
Os01g0934300	Flowering-time protein isoform beta
Os01g0658900	G-box binding factor 1
Os03g0640800	HD-Zip protein (Homeodomain transcription factor) (ATHB-14)
Os03g0745000	Heat shock factor (HSF)-type, DNA-binding domain containing protein
Os02g0232000	Heat shock transcription factor 29 (Fragment)
Os03g0854500	Heat shock transcription factor 31 (Fragment)
Os06g0603000	Heme oxygenase 1 (Fragment)
Os02g0147800	Homeo protein (Fragment)
Os01g0818400, Os03g0109400, Os04g0541700, Os04g0548700	Homeobox domain containing protein
Os05g0129700	Homeobox protein rough sheath 1
Os03g0188900	Homeobox-leucine zipper protein ATHB-6 (Homeodomain transcription factor ATHB-6) (HD-ZIP protein ATHB-6)
Os09g0528200	Homeodomain leucine zipper protein (Fragment)
Os03g0224700	HSP protein (Fragment)
Os03g0673000	Knotted1-type homeobox protein OSH10 (Fragment)
Os03g0727200	Knotted1-type homeobox protein OSH3
Os01g0201700	MADS box protein
Os06g0712700	MADS-box protein SPW1
Os05g0437700	OSE2-like protein (Fragment)
Os02g0797100, Os04g0547600, Os04g0610400, Os05g0549800, Os06g0691100, Os07g0227600, Os08g0408500, Os08g0521600, Os11g0129700	Pathogenesis-related transcriptional factor and ERF domain containing protein
Os01g0883100	PISTILLATA-like MADS box protein
Os01g0174000	Protein HY5 (AtbZIP56)
Os06g0712600	Protein of unknown function DUF702 family protein
Os02g0649300	Short highly repeated, interspersed DNA (Fragment)
Os06g0252300	TF-like protein (Fragment)
Os01g0899800, Os06g0657500	ANT (Ovule development protein aintegumenta)
Os03g0341000	AP2 domain containing protein RAP2.2 (Fragment)
Os03g0191900	AP2 domain family transcription factor homolog (AP2 domain transcription factor)
Os09g0369000	AP2 domain transcription factor RAP2.3 (Related to AP2 protein 3)
Os02g0657000	AP2 domain-containing protein Rap211
Os09g0423800	AP2-1 protein (Fragment)
Os02g0546600	AP2-domain DNA-binding protein
Os04g0539500	AtGATA-3 protein (GATA transcription factor 3)
Os03g0313100, Os08g0442400	BABY BOOM
Os08g0472400, Os09g0456700	Conserved hypothetical protein
Os01g0200300, Os03g0231000	Hypothetical protein

*The chromosomal distribution of the positively expressed genes is shown in Figure S2*.

Among the 70 identified WRKY genes in rice and *Arabidopsis* (Goff et al., 2002; Dong et al., 2003), transcripts of 12 TFs were found to be salt stress responsive in this study (Table 6 and Table S4a2). Of these, *OsWRKY6* (Os03g0798500) and *OsWRKY42* (Os02g0462800) were found to be low phosphorus (Chen et al., 2009) and low boron (Kasajima et al., 2010) responsive, *OsWRKY24* (Os01g0826400) stress responsive (Wei et al., 2013); *OsWRKY34* (Os01g0665500 and Os07g0583700) cold responsive (Zou et al., 2010); *OsWRKY34* (Os02g0265200) heat responsive; all in *Arabidopsis* (Li et al., 2010). However, the TFs WRKY31 (Os01g0750100), WRKY32 (Os02g0770500), WRKY44 (Os08g0276200), WRKY49 (Os01g0730700), WRKY63 (Os06g0158100), and WRKY71 (Os02g0181300) were not reported earlier.

The other important TFs found to be salt responsive in this study include CBF like protein, E2F protein, ethylene responsive TFs, heat shock, and MADS-box proteins and AP2 domain containing proteins (Table 6 and Table S4a2). Chromosome 1, 2, and 3 contain most of the genes while no genes were located in chromosome 10 and 12 (Figure S2). Some of these TFs are reported to play roles in abiotic and biotic stress tolerance in rice and other crops; however, a detailed investigation of the previously unidentified TFs will provide valuable information in explaining salinity tolerance mechanisms in rice.

### Translation factor activity

The regulation of translation, that facilitates the selective synthesis of required proteins, is one of the versatile strategies plants have evolved to cope with the environmental stresses. Generally, in eukaryotes, eukaryotic initiation factor (*eIF4E*) recognizes the 5′-cap structure of mRNAs to begin the canonical cap-dependent translation. The *eIF4G* and *eIF4A* then interacts with *eIF4E* to form the *eIF4F* (cap-binding complex) and the factors *eIF4B, eIF3, eIF1*, and *eIF1A* are subsequently recruited that ultimately regulates translation (Echevarría-Zomeño et al., 2013). Under stresses, the eukaryotic cells usually inhibit this translation initiation that affects the activity of initiation factor *eIF2* and *eIF4E*, a mechanism mainly unknown in plants (Wek et al., 2006; Muñoz and Castellano, 2012). In this study, among the significantly positively and negatively expressed genes, 36 and 26 genes, respectively were found to enrich the translation factor activity (Table 7, Table S4a2; Figure S3).

**Table 7:** **Lists of positively (a) and negatively (b) expressed transcripts (for shoot Na^+^/K^+^) that significantly enriched the translation factor activity in wide natural variation of rice genotypes**.

**Name**	**Annotation**
**(a) Positively expressed transcripts (36)**	
Os02g0146600	Eukaryotic initiation factor 4A (eIF4A) (eIF-4A)
Os03g0566800	Eukaryotic initiation factor 4A-3 (eIF4A-3) (eIF-4A-3)
Os05g0566500	Eukaryotic translation initiation factor 3 subunit 7 (eIF-3 zeta) (eIF3d) (p66)
Os07g0124500	Eukaryotic translation initiation factor 3 subunit 8 (eIF3 p110) (eIF3c)
Os07g0555200	Eukaryotic translation initiation factor 4G
Os07g0597000	Eukaryotic translation initiation factor 5A (eIF-5A)
Os03g0758800	Eukaryotic translation initiation factor 5A-2 (eIF-5A) (eIF-4D)
Os12g0507200	Eukaryotic translation initiation factor 5A-2 (eIF-5A) (eIF-4D)
Os05g0592600	Initiation factor 2 family protein
Os02g0101100	Initiation factor 3 family protein
Os05g0575300	Translation initiation factor IF-2, chloroplast precursor (PvIF2cp)
Os02g0557600, Os05g0498400	Translation initiation factor SUI1 family protein
Os04g0237300	DNA-directed RNA polymerase alpha chain (EC 2.7.7.6) (PEP)
Os03g0851100	Eftu
Os03g0196900	TFIIB-related protein (Fragment)
Os11g0166800	Transcription elongation factor S-II, N-terminal domain containing protein
Os03g0441000	Transcription initiation factor TFIID component TAF4 domain containing protein
Os01g0846900	Transcription initiation factor TFIID domain containing protein
Os07g0662500	Elongation factor 1-beta' (EF-1-beta')
Os06g0571400	Elongation factor 1-gamma (EF-1-gamma) (eEF-1B gamma)
Os01g0742200	Elongation factor EF-2 (Fragment)
Os03g0565500	Elongation factor G 1, mitochondrial precursor (mEF-G-1)
Os02g0456200	G1 to S phase transition protein 1 homolog
Os01g0528000, Os01g0652800 Os01g0655400, Os06g0688100	Hypothetical protein
Os01g0229100, Os02g0122300	Conserved hypothetical protein
Os02g0812400	Nucleotidyl transferase domain containing protein
Os05g0277300	Peptide chain release factor 1
Os07g0503700	Proteasome component region PCI domain containing protein
Os01g0887200	Winged helix DNA-binding domain containing protein
Os06g0597400	ZLL/PNH homologous protein
Os04g0168100	Zn-finger, C2H2 type domain containing protein
**(b) Negatively expressed transcripts (26)**	
Os04g0533000	ATP-dependent RNA helicase p54 (Xp54)
Os05g0227700, Os07g0191700	Conserved hypothetical protein
Os03g0177400, Os03g0178000	EF-1 alpha
Os11g0116400	Elongation factor P (EF-P)
Os12g0541500	Elongation factor Ts (EF-Ts)
Os07g0614500	Elongation factor 1-beta (EF-1-beta)
Os02g0220500, Os02g0220600	Elongation factor 1-gamma (EF-1-gamma) (eEF-1B gamma)
Os02g0300700	Eukaryotic translation initiation factor 1A (EIF-1A) (EIF-4C)
Os07g0681000	Eukaryotic translation initiation factor 2 beta subunit (eIF-2-beta) (P38)
Os01g0120800	Eukaryotic translation initiation factor 3 subunit 10 (eIF-3 theta)
Os07g0167000	Eukaryotic translation initiation factor 3 subunit 6 (eIF-3 p48) (eIF3e)
Os01g0970400	Eukaryotic translation initiation factor 4E-1 (eIF4E-1) (eIF-4E-1)
Os12g0607100	Histone-lysine N-methyltransferase, H3 lysine-9 specific (EC 2.1.1.43)
Os02g0794400	Initiation factor three family protein
Os05g0107700	Transcription initiation factor IIA gamma chain (TFIIA-gamma)
Os07g0639800	Translation initiation factor IF6 family protein
Os06g0338900	Nucleotidyl transferase domain containing protein
Os05g0277300	Peptide chain release factor 1
Os02g0606100	Quinoprotein amine dehydrogenase, beta chain-like domain containing protein
Os02g0641800	RNA helicase
Os12g0165700	Transcription factors TFIIS, elongin A, CRSP70, conserved domain containing protein
Os01g0772200, Os10g0397200	Winged helix DNA-binding domain containing protein

*The chromosomal distribution of the positively expressed genes is shown in Figure S3*.

Among the up-regulated genes the most noticeable fall under the category of translation initiation factors such as Os02g0146600 (eIF4A), Os03g0566800 (eIF4A-3), Os05g0566500 (eIF-3 zeta), Os07g0124500 (eIF3 p110), Os07g0555200 (eIF4G), Os07g0597000 (eIF-5A), Os03g0758800, and Os12g0507200 (eIF-5A) (eIF-4D), Os05g0592600 (eIF 2 family protein), Os02g0101100 (eIF 3 family protein) and Os02g0557600, and Os05g0498400 (IF SUI1 family protein); transcription elongation factors such as Os03g0196900 (TFIIB), Os11g0166800 (TFS-II), Os03g0441000 and Os01g0846900 (TFIID), Os07g0662500 (EF-1-beta'), Os06g0571400 (EF-1-gamma), Os01g0742200 (EF-2), and Os03g0565500(mEF-G-1) etc.

Several elongation factors e.g., Os03g0177400 and Os03g0178000 (EF-1 α), Os11g0116400 (EF-P), Os12g0541500 (EF-Ts), Os07g0614500 (EF-1-beta), and Os02g0220500 and Os02g0220600 (EF-1-gamma) etc., and several translation initiation factors e.g., Os02g0300700 (EIF-1A), Os07g0681000 (eIF-2-beta) (P38), Os01g0120800 (eIF-3 theta), Os07g0167000 (eIF-3 p48), Os01g0970400 (eIF4E-1), Os02g0794400 (IF-3 family protein), Os05g0107700 (TFIIA-gamma), and Os07g0639800 (IF6 family protein) etc., were also found to be negatively expressed.

### SNAP receptor and chaperone activity

SNAP receptor activity is regulated by a super family of proteins known as SNAREs [soluble N-ethylmaleimide-sensitive factor (NSF) attachment protein receptors] that act as a marker to identify a membrane and selectively interact with SNAREs on other membrane surfaces to mediate membrane fusion thus providing a continuous flux of membranes via transport vesicles. This vesicle traffic is believed to be involved in cell homeostasis, growth, and development of plants (Tyrrell et al., 2007; Kim and Brandizzi, 2012). In this study, among the genes that are negatively expressed for shoot Na/K, six genes that significantly enriched the SNAP receptor activity in wide rice genotypes under salt stress were identified (Table S4a2). The bet like SNARE- *AtBS14a* (Os02g0820700 and Os08g0563300) that were found to be significant was reported to control cell growth in *Arabidopsis* (Tai and Banfield, 2001). The syntaxin identified is *AtSYP52* (encoded by Os02g0119400) was very recently described to act as t-SNARE when distributed in membrane TGN/PVC and plays a putative inhibitory role when present on the tonoplast in *Arabidopsis* (Benedictis et al., 2013). Another syntexin, *OSM1*/*SYP61*, was also reported to be involved in osmotic stress tolerance in Arabidopsis (Zhu et al., 2002). However, three other syntexins encoded by Os07g0164300, Os01g0254900, and Os06g0168500 that were found to be significant in this study might be novel syntexin and it would be of interest to know their specific role in future.

Chaperones are proteins involved in non-covalent folding or unfolding of other proteins and are believed to be expressed in response to high temperature and other cellular stresses. Yamada et al. (2002) identified a cytosolic chaperonin-containing TCP-1α (CCTα) homolog that displayed enhanced salt tolerance in the mangrove plant, *Bruguiera sexangula*. In this study, six transcripts that significantly enriched chaperone binding activity under salt stress were identified. These are GrpE type 2 (Os08g0338700), GrpE protein family protein (Os04g0431100 and Os09g0284400), DRF2 (Os12g0456200) and one protein of unknown function (Os12g0456200) and another conserved hypothetical protein (Table S4a2).

### Interacting network of genes under salt stress

**Figure 2:**
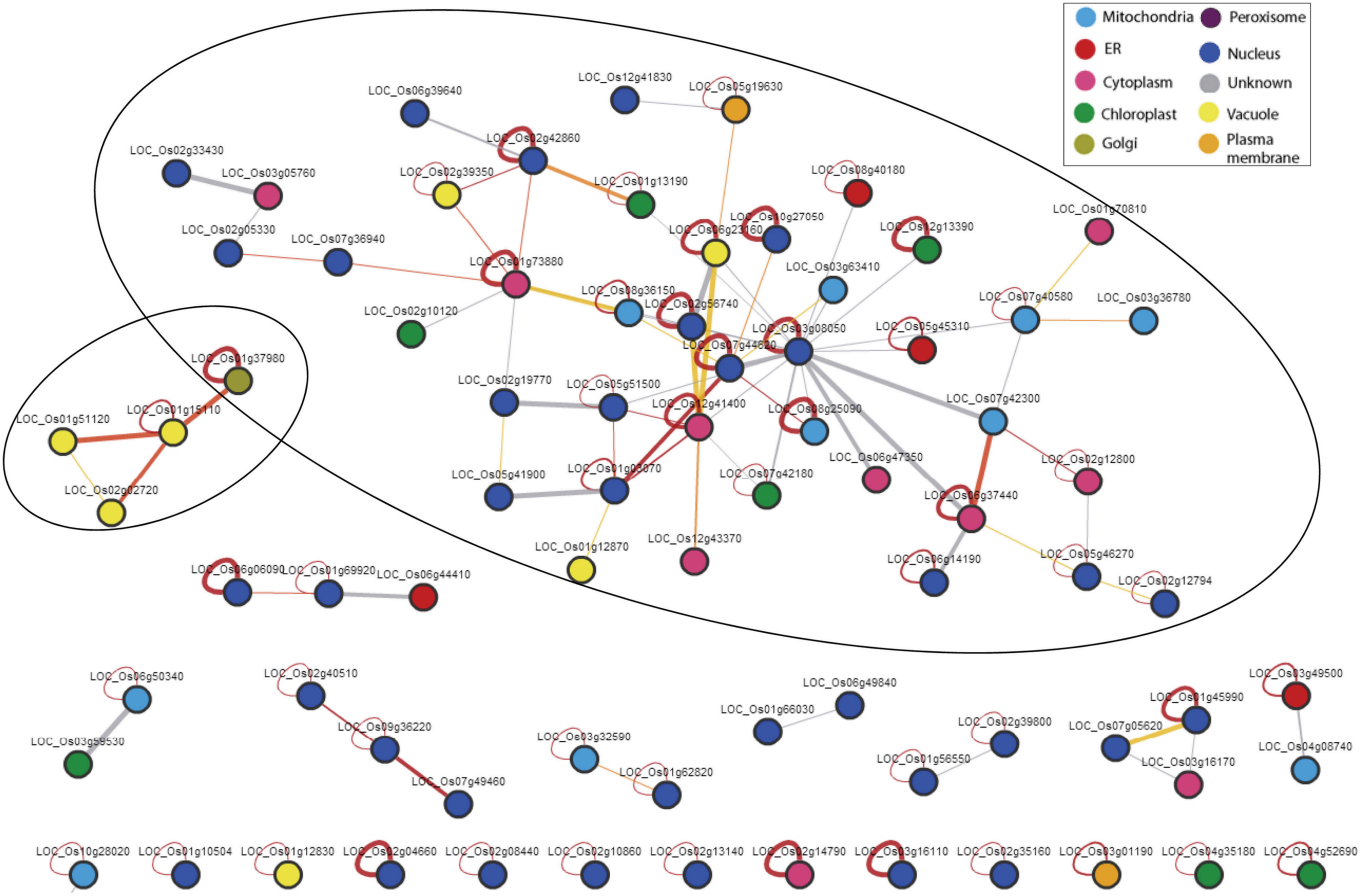
**Regulatory networks of all the 578 genes that significantly enriched the molecular functional categories under salt stress in wide natural variation of rice genotypes**. The web based tool “Rice Interactions Viewer” (http://bar.utoronto.ca/interactions/cgi-bin/rice_interactions_viewer.cgi) were used to predict the interactions.

The interactive networks analysis of all the significant genes (in total 578), as determined by SEA, revealed two networks (Figure 2). In the larger network, LOC_Os03g08050 (Os03g0177400) seemed to be the central protein which encodes for “Protein elongation factor (EF-1 alpha).” Most of the proteins in this network seem to be localized mainly in nucleus (blue), cytoplasm (pink), and mitochondria (light blue) and encode mainly for the translation factors such as LOC_Os02g56740 (protein translation initiation factor eIF-2B subunit epsilon, LOC_Os05g51500 (protein eukaryotic translation initiation factor 5B), LOC_Os07g44620 (protein eukaryotic translation initiation factor 6), LOC_Os05g41900 (protein translation initiation factor SUI1), LOC_Os02g19770 (protein eukaryotic translation initiation factor 1A), LOC_Os07g36940 (protein eukaryotic translation initiation factor 4G) etc., and transcription factors such as LOC_Os06g14190 (protein NF-X1-type zinc finger protein). On the contrary, only a few proteins are located in the chloroplast (green) and plasma membrane (brown) and vacuole (yellow). The proteins localized in the chloroplast (green) are mainly catalytic proteins such as LOC_Os12g13390 (protein aspartyl aminopeptidase, putative), LOC_Os01g13190 (protein histidinol dehydrogenase, chloroplast precursor), LOC_Os02g10120 (protein lipoxygenase) and LOC_Os07g42180 (protein exportin 1). Proteins expressed in the vacuole includes LOC_Os06g23160 (protein bacterial transferase hexapeptide domain containing protein), LOC_Os01g12870 (protein eukaryotic translation initiation factor 3 subunit E-interacting protein), and LOC_Os02g39350 (protein eukaryotic translation initiation factor 2A) etc.

In the smaller network, all the proteins are SNAREs type proteins (SNAP receptor activity) with three localized in the vacuole encoding syntaxin (LOC_Os01g15110), vesicle transport v-SNARE protein (LOC_Os01g51120), and SNARE domain containing protein (LOC_Os02g02720) and one localized in the plasma membrane encoding vesicle transport v-SNARE protein (LOC_Os01g37980).

These finding probably points toward the hypothesis that in the wide natural gene pool, transcription and translation factor genes are the main regulators under salt stress and these genes are probably the initial defense response that co-regulates in an interactive manner which ultimately cascades to induce the downstream genes that are essential for adaptation to environmental stresses.

## Conclusion

The genes that are identified here provide a synergistic global salinity response picture representing the mechanisms that are active in the wide natural variation of rice genotypes which may not be referred back to individual genotypes in the context of this study (as gene expression responses of all the genotypes were analyzed together) but might be of interest to look at their specific role in individual rice genotypes in future. Besides, the gene expression profile of these genes in different cell or tissue types may also needed to be studied in a way to verify the pathways they are involved in via *in situ* analysis that will help to better understand their roles in salinity tolerance mechanism. Nonetheless, the products of these genes may hold the key to the evolutionary adaptive mechanism to cope with saline environments. Several of the identified genes were reported before either in rice or in other crop species, however, the novel genes and the genes with unknown function may enhance our understanding of stress adaptation once the role of these genes are functionally verified.

## Author contributions

MH, GB, JP, and BF jointly conceptualized and designed the project. MH has conducted all experiments, generated, and analyzed the data and prepared the draft script. GS helped in lab work, analysis and in finalizing the draft. JP, GB, and BL supervised the work, interpreted the results, and finalized the manuscript.

### Conflict of interest statement

The authors declare that the research was conducted in the absence of any commercial or financial relationships that could be construed as a potential conflict of interest.

## Supplementary material

Figure S1 **The chromosomal distribution of the 107 positively expressed genes across the 12 chromosomes that significantly enriched the “signal transducer activity.”** The chromosome map is obtained by submitting the list of genes to the web based “Chromosome Map Tool” http://viewer.shigen.info/oryzavw/maptool/MapTool.do (see Materials and Methods Section for details).

Figure S2 **The chromosomal distribution of the 81 negatively expressed genes across the 12 chromosomes that significantly enriched the “transcription factor activity”**.

Figure S3 **The chromosomal distribution of the 36 positively (A) and 26 negatively (B) expressed genes for shoot Na/K that significantly enriched translation factor activity in wide natural rice genotypes under salt stress**.

Table S1 **(a–n)** Positively and negatively regulated significant probes for each of the 14 morpho-physiological traits in the eight rice genotypes representing wide natural variation in terms of salinity tolerance and genetic diversity under unstressed condition determined by Significance Analysis of Microarrays (SAM). Significant probes were selected based on the criteria of FDR <5% (*q*-value) and fold score >2.0 (*d*) and are presented along with the corresponding Locus ID and annotation.

Table S2 **(a–n)** Positively and negatively regulated significant probes for each of the 14 morpho-physiological traits in the eight rice genotypes representing wide natural variation in terms of salinity tolerance and genetic diversity under stressed condition determined by Significance Analysis of Microarrays (SAM). Significant probes were selected based on the criteria of FDR <5% (*q*-value) and fold score >2.0 (*d*) and are presented along with the corresponding Locus ID and annotation.

Table S3 **Lists of probes along with the corresponding functional annotation and fold scores that commonly regulated for the trait Biomass under unstressed and stressed conditions in wide natural variation of rice genotypes**.

Table S4 **Lists of significant GO categories of molecular functions and biological processes along with GO terms, *p*-values, FDR values, GO flash charts and schematic diagrams under unstressed condition for differentially regulated (positive and negative) genes for different morpho-physiological traits (determined by SEA analysis)**.

Table S5 **Lists of significant GO categories of molecular functions and biological processes along with GO terms, *p*-values, FDR values, GO flash charts and schematic diagrams under stressed condition for differentially regulated (positive and negative) genes for different morpho-physiological traits (determined by SEA analysis)**.
