# Supplementary figures and images for: Trait Specific Expression Profiling of Salt Stress Responsive Genes in Diverse Rice Genotypes as Determined by Modified Significance Analysis of Microarrays
Source: Front Plant Sci. 2016 May 3;7:567. doi: 10.3389/fpls.2016.00567 (PMC4853522; doi:10.3389/fpls.2016.00567)

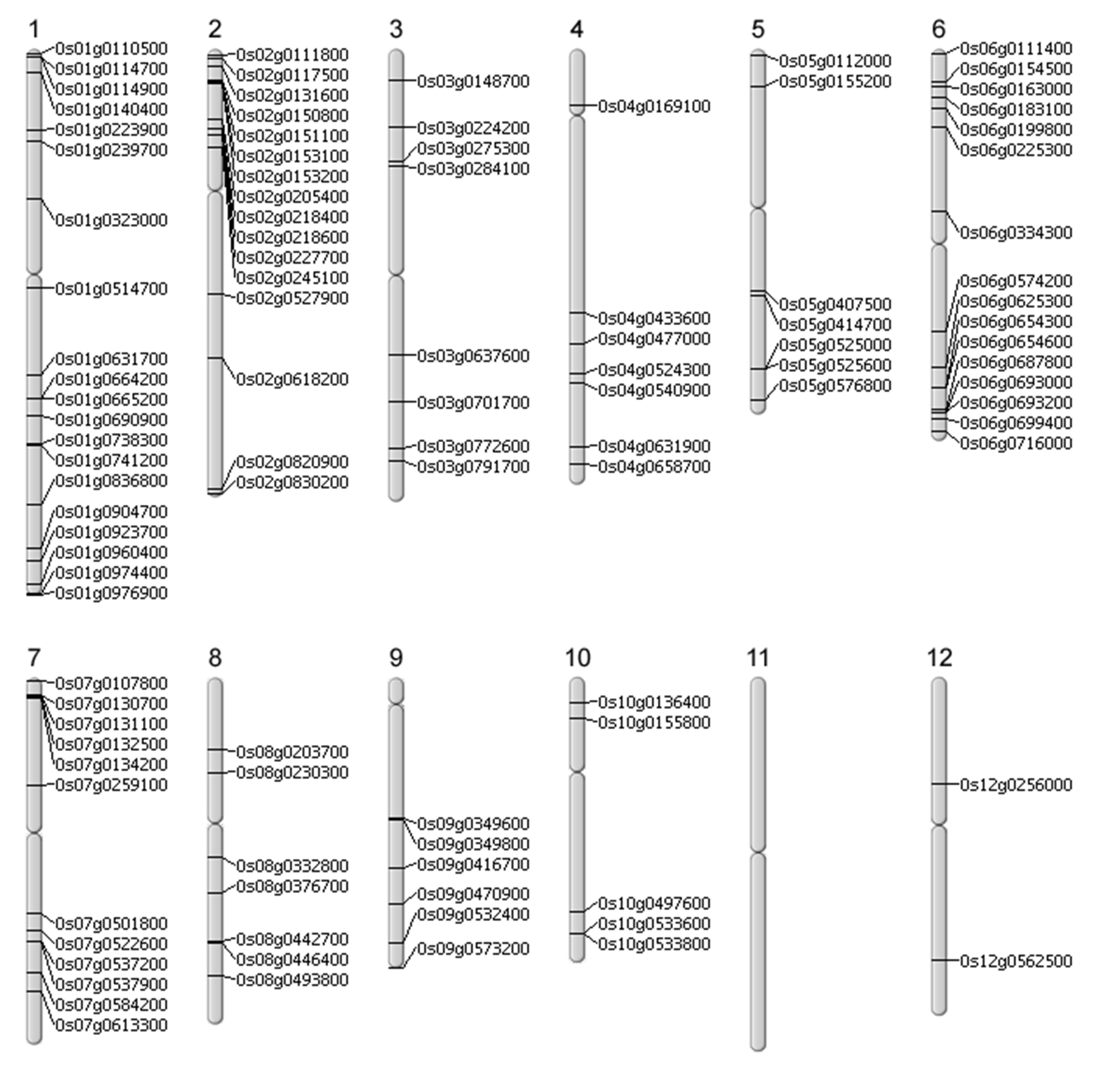

Supplement: Figure S1 — The chromosomal distribution of the 107 positively expressed genes across the 12 chromosomes that significantly enriched the “signal transducer activity.” The chromosome map is obtained by submitting the list of genes to the web based “Chromosome Map Tool” http://viewer.shigen.info/oryzavw/maptool/MapTool.do (see Materials and Methods Section for details). [file Image1.TIF]

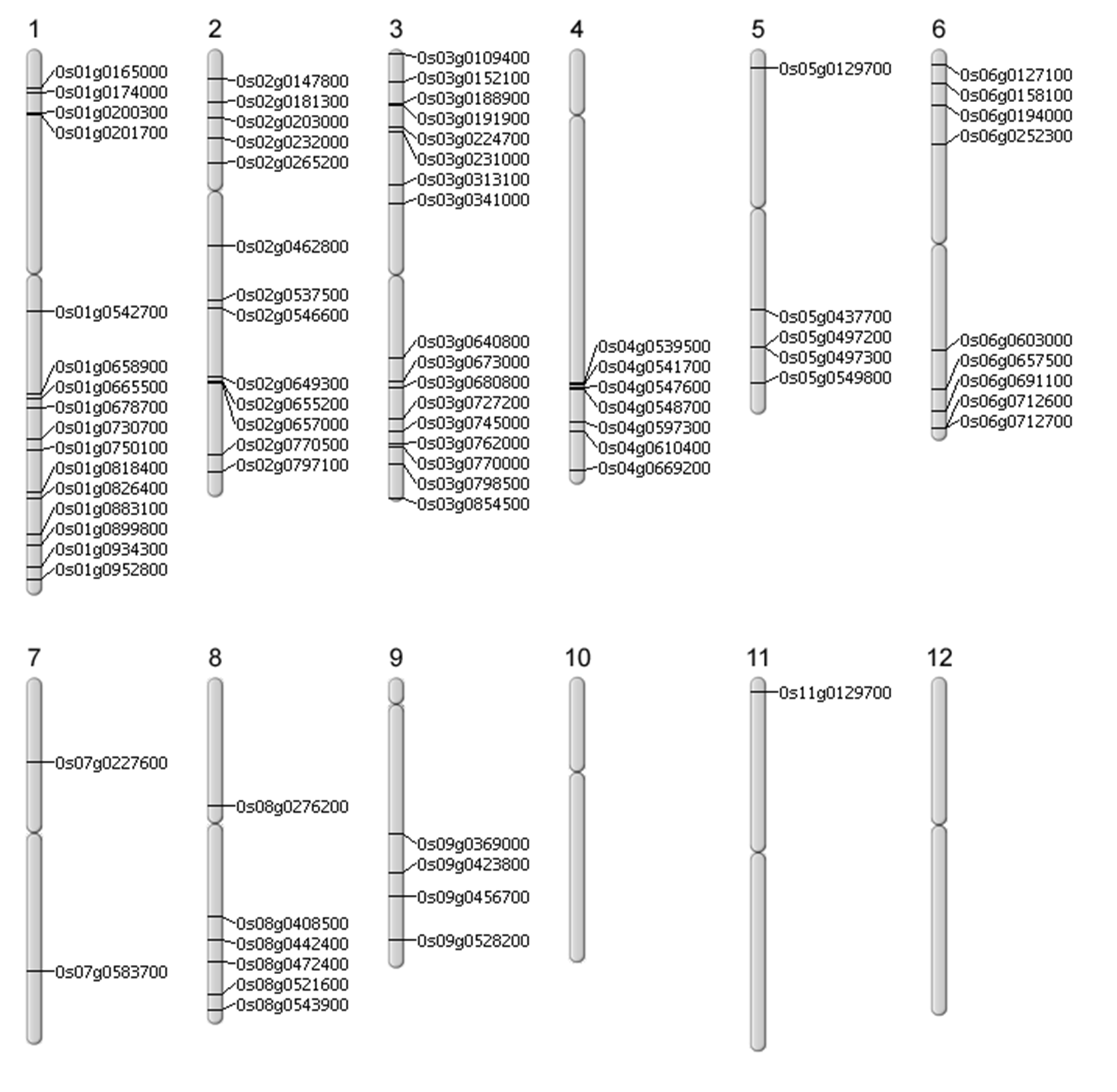

Supplement: Figure S2 — The chromosomal distribution of the 81 negatively expressed genes across the 12 chromosomes that significantly enriched the “transcription factor activity”. [file Image2.TIF]

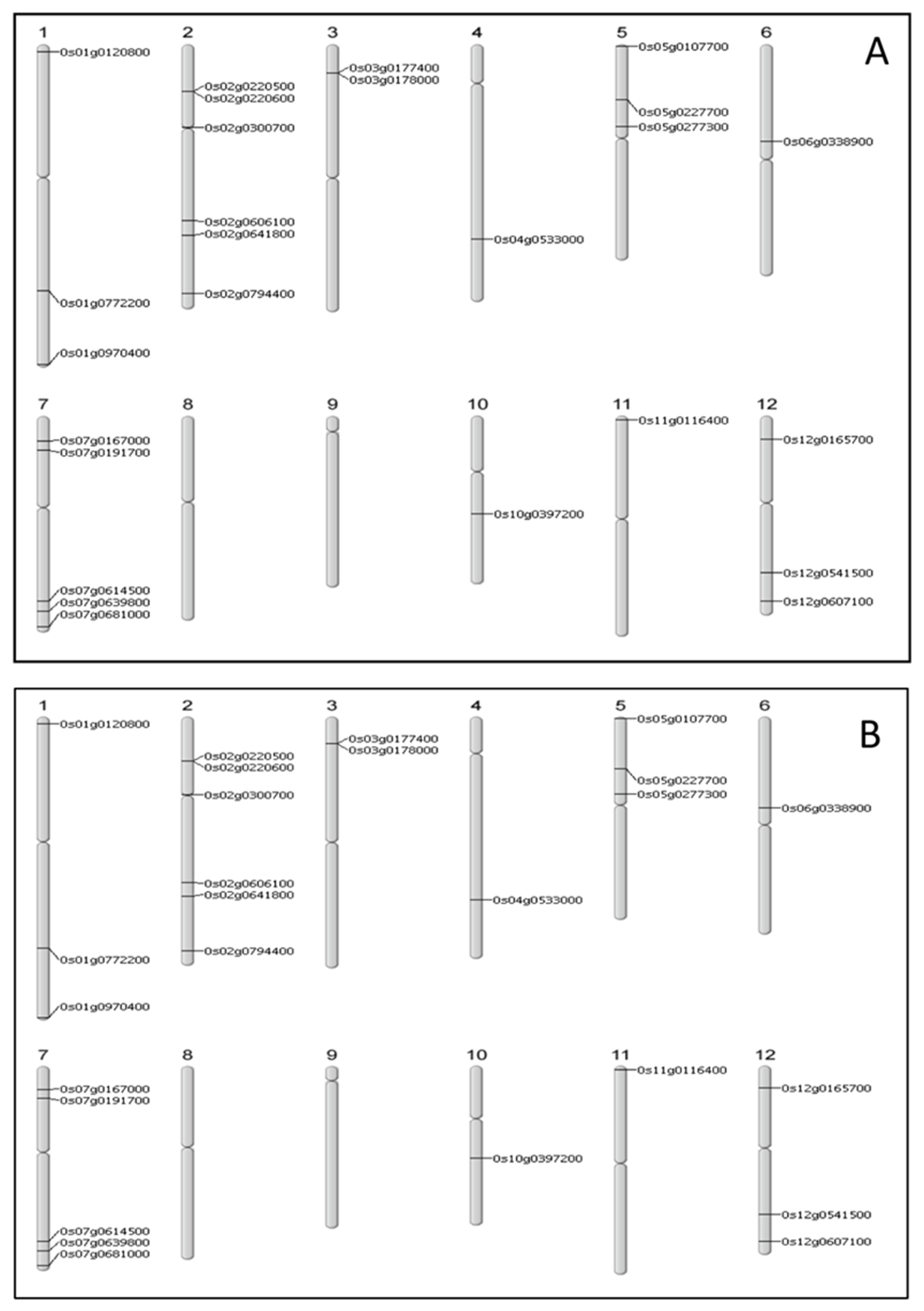

Supplement: Figure S3 — The chromosomal distribution of the 36 positively (A) and 26 negatively (B) expressed genes for shoot Na/K that significantly enriched translation factor activity in wide natural rice genotypes under salt stress. [file Image3.TIF]
